# Scallop protein with endogenous high taurine and glycine content prevents high-fat, high-sucrose-induced obesity and improves plasma lipid profile in male C57BL/6J mice

**DOI:** 10.1007/s00726-014-1715-1

**Published:** 2014-03-23

**Authors:** Hanne Sørup Tastesen, Alison H. Keenan, Lise Madsen, Karsten Kristiansen, Bjørn Liaset

**Affiliations:** 1 Department of Biology, University of Copenhagen, Ole Maaløes Vej 5, 2200 Copenhagen, Denmark; 2 National Institute of Nutrition and Seafood Research, Postbox 2029, Nordnes, 5817 Bergen, Norway; 3 Massachusetts General Hospital, Center for Computational and Integrative Biology, 185 Cambridge Street, Boston, MA 02114 USA

**Keywords:** Cholesterol, Diet-induced obesity, Plasma lipid profile, Protein, Seafood, Taurine

## Abstract

High-protein diets induce alterations in metabolism that may prevent diet-induced obesity. However, little is known as to whether different protein sources consumed at normal levels may affect diet-induced obesity and associated co-morbidities. We fed obesity-prone male C57BL/6J mice high-fat, high-sucrose diets with protein sources of increasing endogenous taurine content, i.e., chicken, cod, crab and scallop, for 6 weeks. The energy intake was lower in crab and scallop-fed mice than in chicken and cod-fed mice, but only scallop-fed mice gained less body and fat mass. Liver mass was reduced in scallop-fed mice, but otherwise no changes in lean body mass were observed between the groups. Feed efficiency and apparent nitrogen digestibility were reduced in scallop-fed mice suggesting alterations in energy utilization and metabolism. Overnight fasted plasma triacylglyceride, non-esterified fatty acids, glycerol and hydroxy-butyrate levels were significantly reduced, indicating reduced lipid mobilization in scallop-fed mice. The plasma HDL-to-total-cholesterol ratio was higher, suggesting increased reverse cholesterol transport or cholesterol clearance in scallop-fed mice in both fasted and non-fasted states. Dietary intake of taurine and glycine correlated negatively with body mass gain and total fat mass, while intake of all other amino acids correlated positively. Furthermore taurine and glycine intake correlated positively with improved plasma lipid profile, i.e., lower levels of plasma lipids and higher HDL-to-total-cholesterol ratio. In conclusion, dietary scallop protein completely prevents high-fat, high-sucrose-induced obesity whilst maintaining lean body mass and improving the plasma lipid profile in male C57BL/6J mice.

**Electronic supplementary material:**

The online version of this article (doi:10.1007/s00726-014-1715-1) contains supplementary material, which is available to authorized users.

## Introduction

As obesity and a range of co-morbidities, such as type 2 diabetes mellitus and cardiovascular disease, have become major public health challenges worldwide, research into prevention and alleviation of obesity has intensified. Proteins induce greater satiety than carbohydrates and fat in human subjects (Rolls et al. 1988; Weigle et al. 2005) and rats (Bensaïd et al. 2002). Consequently, increasing the proportion of protein in the diet potentially decreases energy consumption and ultimately may contribute to weight loss. Indeed, in human subjects an increase in dietary protein level, at the expense of fat or carbohydrate, has been shown to promote weight loss (Weigle et al. 2005) and to improve body composition and short-term weight maintenance (Westerterp-Plantenga et al. 2004). It is still uncertain whether the beneficial effects of increasing protein in the diet are maintained over time in humans (Due et al. 2004), whereas in mice we have previously shown that increasing dietary protein (casein) at the expense of carbohydrates (sucrose) reduces diet-induced obesity over time (Hao et al. 2012; Ma et al. 2011; Madsen et al. 2008). Apart from quantity, the quality of dietary proteins is of significance in the prevention of obesity. Prospective cohort studies have demonstrated that consumption of fish as a part of healthy diet is associated with lower body weight (Schulze et al. 2006; Shubair et al. 2005) and randomized controlled studies show that the inclusion of fish in energy-restricted diets resulted in greater weight loss compared to control diets without seafood (Thorsdottir et al. 2007; Ramel et al. 2009). In addition, incorporation of a daily fish meal into a weight-loss regimen was more effective than either fish consumption or weight loss alone at improving glucose-insulin metabolism and dyslipidemia (Mori et al. 1999). The beneficial properties of seafood have mainly been ascribed to the marine n-3 long-chain PUFAs, but other factors, e.g., vitamin D, selenium, iodine and taurine, have been reported to also contribute to the benefits associated with consumption of seafood (Lund 2013). Insulin resistant subjects showed improved insulin sensitivity after 4 weeks on a cod-based diet compared with subjects ingesting a meat-based diet (Ouellet et al. 2007); an effect possibly associated with the cod-based diet’s ability to reduce C-reactive protein levels (Ouellet et al. 2008). Also, a daily supplement of cod protein resulted in improved body composition and decreased blood LDL-cholesterol in overweight adults (Vikøren et al. 2013). Seafood protein is characterized by high levels of taurine compared to terrestrial protein sources (Spitze et al. 2003). Supplementation of taurine in the diet or drinking water has been shown to prevent diet-induced weight gain and adiposity in rodents (Camargo et al. 2013; Nardelli et al. 2011) and to improve blood lipid profiles in rodents (Fukuda et al. 2011; Murakami et al. 1998; Nardelli et al. 2011; Yokogoshi et al. 1999) and human subjects (Zhang et al. 2004). To the best of our knowledge no studies have been published evaluating the effects of dietary protein sources with endogenously varying contents of taurine on the development of diet-induced obesity and related diseases. We hypothesize that an increase in dietary taurine through consumption of seafood protein might prevent diet-induced fat accretion and improve plasma lipid profile in a dose-dependent way. To test this hypothesis we fed male C57BL/6J mice high-fat, high-sucrose diets with protein containing increasing endogenous levels of taurine, i.e., chicken fillet, cod fillet, white crab meat and scallop muscle, as the sole protein source for 6 weeks. C57BL/6J mice are prone to diet-induced obesity (West et al. 1992) and are thus widely used in research on obesity, glucose intolerance, insulin resistance, dyslipidemia and related disease states. A high-fat, high-sucrose diet was used in this study as it has been shown to be more obesogenic than high fat (Surwit et al. 1995) and high-fat, high-protein feeding (Ma et al. 2011). We found that the scallop diet prevented diet-induced obesity without affecting lean body mass. Furthermore, the plasma lipid profile was improved in scallop-fed mice compared to chicken, cod and crab-fed mice.

## Materials and methods

### Ethical statement

The animal experiments were approved by the National Animal Health Authorities (Norwegian approval identification number 2497). Care and handling were performed in accordance with local institutional rules and the ethical standards laid down in the 1964 Declaration of Helsinki and its later amendments. No adverse events were observed.

### Animal studies

Male C57BL/6JBomTac mice, weighing approximately 25 g, were obtained from Taconic Europe (Ejby, Denmark). The mice were housed individually and kept on a 12:12 h light–dark cycle at thermoneutrality (28 ± 1 °C). To obtain both fasted and non-fasted (randomly fed) blood plasma at the termination, two identical studies were carried out in parallel 3 weeks apart, i.e., experiment 1 (Expt. 1) and experiment 2 (Expt. 2), respectively. Upon arrival, the mice were let to acclimatize for 5 days and were then assigned to the different experimental diet groups (*n* = 8 mice per group) by body mass. The mice were fed ad libitum, and the feed intake as well as body mass was measured throughout the feeding period. After 4 weeks on the experimental diets, the mice were kept in cages with paper lining, instead of the standard wood-chip bedding, for 48 h with the purpose of collecting feces (Expt. 1). Following 6 weeks of feeding, the mice were terminated by inhalation of Isoflurane (4 %, Baxter, IL, USA) followed by cardiac puncture exsanguination either in the overnight fasted (Expt. 1) or in the non-fasted (Expt. 2) state. At termination selected tissues were dissected and weighed. Total fat mass was compared between the groups by comparing the combined mass of dissected fat depots; inguinal white adipose tissue (iWAT), interscapular brown adipose tissue (iBAT), mesenteric white adipose tissue (MeWAT), epididymal white adipose tissue (eWAT) and perirenal and retroperitoneal white adipose tissue (p/rWAT). Lean mass was compared between the groups by comparing the mass of dissected heart, tibialis anterior and soleus. Unless otherwise stated data shown in this paper is from Expt. 2.

### Experimental diets

Based on a high-fat, high-sucrose background diet of Lavigne et al. (2001), four different isonitrogenous and isoenergetic experimental diets were made containing protein from four different sources with varying taurine concentrations; chicken breast fillets (chicken, taurine in diet 0.3 g/kg), wild caught cod fillets (cod, taurine in diet 1.7 g/kg), white crab meat (crab, taurine in diet 2.4 g/kg) and Canadian scallop muscles (scallop, taurine in diet 12.9 g/kg). A fifth group of mice fed a low-fat diet (LF, OpenSource Diet no. D12450B, Research Diets Inc., NJ, USA) with casein as the sole protein source was included as a reference group only and was not included in the statistical analyses. The protein sources were the sole source of AAs and no supplementary AAs were added to the diets. The protein sources had differing endogenous ash content, i.e., the residue left after combustion reflecting mineral content, which may influence energy density (J/g). To balance dietary ash content, varying amounts of potassium chloride (KCl) were added to the chicken (13.4 g/kg), cod (10.8 g/kg) and scallop (5.9 g/kg) diets (Table 1). Potassium chloride was used to avoid elevations in blood pressure. The final compositions of the diets are listed in Tables 1, 2 and Supplemental Table 1 (Online Resource 1). Feed efficiency was calculated as body mass gain per energy intake as follows (Eq. 1):

**Table 1:** Composition of the experimental diets

	LF^a^	Chicken^b^	Cod^c^	Crab^d^	Scallop^e^
Composition (g/kg)					
Casein	200	–	–	–	–
Chicken	–	231	–	–	–
Cod	–	–	228	–	–
Crab	–	–	–	253	–
Scallop	–	–	–	–	248
KCl	–	13.4	10.8	–	5.9
Sucrose	350	226	232	218	217
Cellulose	50	50	50	50	50
Lard	20	198	198	198	198
Vegetable oil^f^	25	198	198	198	198
Mineral mix^g^	10	67	67	67	67
Vitamin mix^h^	10	14	14	14	14
Choline bitartrate	2	2	2	2	2
Butylated hydroxytoluene	–	0.4	0.4	0.4	0.4
Analyzed (g/kg)					
Crude protein (Nx6.25)	167	199	206	199	207
Fat	46	407	400	405	402
Cholesterol	0.06	0.83	0.84	1.10	0.51
Ash	30	71	74	76	76
Gross energy (kJ/g)	17.7	25.8	25.6	25.8	25.4

^a^OpenSource diet no. D12450B (Research Diets, Inc., NJ, USA)

^b^Chicken breast fillets (Ytterøykylling AS, Ytterøy, Norway)

^c^Cod fillets (Wild caught in the Northeastern Atlantic)

^d^White crab meat (HitraMat AS, Ansnes, Norway)

^e^Canadian scallops (Placopecten magellanicus)

^f^LF: soybean oil. Chicken, cod, crab and scallop: corn oil

^g^LF: Mineral Mix S10026, HFHS: AIN76 mineral mix

^h^LF: Vitamin Mix V100001, HFHS: AIN76 vitamin mix

**Table 2:** Composition of amino acids in the diets

g/kg	LF	Chicken	Cod	Crab	Scallop
Ala	5.3	12.1	12.2	10.9	9.0
Arg	5.6	11.4	10.8	14.6	14.6
Asx	13.4	20.6	22.0	22.1	17.7
Cys	3.2	2.4	2.8	3.1	3.3
Glx	40.2	30.0	29.1	30.7	25.3
Gly	3.1	7.8	8.6	9.3	19.3
His^a^	4.3	6.3	4.0	4.7	2.7
Ile^a^	8.6	9.6	8.8	9.6	7.0
Leu^a^	16.0	16.1	15.7	15.5	12.3
Lys^a^	14.6	19.5	19.1	17.1	14.2
Met^a^	4.5	5.3	6.0	5.8	4.4
Phe^a^	8.3	7.6	7.9	8.6	5.7
Pro	18.1	7.0	6.5	8.0	4.6
Ser	9.9	7.9	8.8	8.8	6.7
Thr^a^	7.3	8.9	8.6	9.7	6.5
Trp	1.8	2.1	2.0	2.0	1.3
Tyr	7.8	5.7	5.9	7.2	4.1
Val^a^	10.8	10.1	9.8	9.8	6.4
OH-Pro	0.0	0.4	0.3	0.1	0.4
Tau	0.0	0.3	1.7	2.4	12.9
EAA	81.6	96.9	92.7	97.2	75.1
BCAA	35.3	35.8	34.3	34.8	25.7
Total	191.6	201.6	200.0	209.4	184.6

*EAA* sum of essential amino acids, *BCAA* sum of branched-chain amino acids (Leu, Ile and Val), *Total* sum of total amino acids

^a^Essential amino acids

1 $${\text{Feed}}\;{\text{efficiency}} = {\text{body}}\;{\text{mass}}\;{\text{gain}}\;({\text{g}})/{\text{energy}}\;{\text{intake}}\;({\text{MJ}}).$$

### Analyses of diet compositions

Energy contents were determined by bomb calorimetry following the manufacturer’s instruction (Parr Instruments, Moline, IL, USA). Fatty acids were extracted from the samples with 2:1 chloroform: methanol (v/v) and internal standard 19:0 methyl ester was added. The samples were filtered, saponified and esterified in 12 % BF_3_ in methanol (v/v). Fatty acid methyl esters were separated on a gas chromatograph (GLC Trace GC 2000, Thermo Scientific, USA) and detected with a flame ionization detector (Thermo Scientific) (Lie and Lambertsen 1991). The fatty acids were identified by retention time using standard mixtures of methyl esters (Nu-Chek-Prep, Elyian, MN, USA) and quantified by the internal standard method. For total amino acid analysis norvaline was added as an internal standard before samples were hydrolyzed in 6 M HCl at 110 ± 2 °C for 22 h. Thereafter, HCl was removed and samples were derivatized with the AccQ-Tag Ultra Derivatization Kit (Waters, USA). Amino acids (AAs) were separated and detected on the ACQUITY UPLC System (Waters, USA). AAs were identified using an amino acid (AA) standard (Pierce Amino Acid Standard H, Thermo Fisher Scientific Inc., IL, USA) to which norvaline, taurine and hydroxy-proline were added. AAs were quantified by internal and external standard regression. Tryptophan was analyzed in the samples after basic hydrolysis with Ba(OH)_2_ for 20 h at 110 ± 2 °C. The samples were pH adjusted to 6.2, separated on a HPLC (Shimadzu 6A/6B) equipped with a SUPELCOSIL™ LC-18 HPLC-column (4.6 mm × 15 cm) and detected in a UV-spectrophotometer (Shimadzu SPD 6A) at 280 nm. Tryptophan was quantified using a standard curve of l-Tryptophan (Sigma T-0254) based on 0.05 and 0.1 mg tryptophan mL^−1^. Total cysteine in the samples was determined after oxidation of cysteine/cystine with 9:1 performic acid (88 %): H_2_O_2_ (30 %) (v/v) to yield cysteic acid. Total cysteine analysis was performed by the Norwegian Institute of Food, Fishery and Aquaculture.

### Total fat in diets and feces

Total fat content was determined gravimetrically after extracting samples with organic solutions. First, samples were mixed with n-heptane, mixed for 10 min and centrifuged. The organic phase was collected. Thereafter, *n*-heptane and 4 M HCl was added to the sample remnant before the mixture was heated to 90 °C for 2 h. After cooling and centrifugation, the organic top fraction was collected. The aqueous bottom fraction including the sample remnant was quantitatively transferred to liquid–liquid extraction column (Chem Elut CE1010, Varian) mixed with Hydromatrix to remove water, and extracted twice with petroleum ether. The eluates were collected and all organic fractions combined, evaporated under vacuum (Evaporator Syncore Analyst, Büchi), dried at 103 °C for 30 min and weighed.

### Nitrogen in diets and feces

The nitrogen content was determined by the Dumas method in a Leco FP-528 nitrogen analyzer (Leco Corp, MI, USA). The crude protein content in the diets was calculated as nitrogen content multiplied by 6.25 (Greenfield and Southgate 2003). The 6.25 conversion factor operates on the underlying assumption that proteins on average contain 16 % nitrogen (100/16 = 6.25).

### Apparent fat and nitrogen digestibility

Feces from the 48 h collection was weighed and analyzed for nitrogen and total fat content. Apparent digestibility was calculated as follows (Eq. 2):

2 $$100 \times ({\text{intake}}\, ( {\text{mg)}} - {\text{fecal}}\;{\text{output}}\,({\text{mg}}))/({\text{intake}}\, ( {\text{mg)}}).$$

### Plasma metabolite measurements

Heparinized plasma was prepared from blood collected by cardiac puncture at the termination and stored at −80 °C prior to analysis. Insulin concentrations were analyzed by DRG Ultrasensitive Mouse Insulin ELISA kit (DRG Diagnostics, Germany). Plasma alanine aminotransferase, total cholesterol, LDL-cholesterol, triacylglycerides (TAG), glucose (MaxMat, France), non-esterified fatty acids (NEFA), HDL-cholesterol, total bile acids (Dialab, Austria), hydroxy-butyrate (OH-butyrate), glycerol (Randox, UK) and lactate (Sentinel Diagnostics, Italy) concentrations were analyzed by conventional kits using a MaxMat PL II analyzer (MAXMAT S.A., Montpellier, France).

### HOMA-IR and QUICKI

The homeostatic model assessment of insulin resistance (HOMA-IR) and the quantitative insulin sensitivity check index (QUICKI) scores were calculated based on overnight fasting plasma glucose and overnight fasting plasma insulin as follows, respectively;

3 $${\text{Glucose}}\;({\text{mmol}}/{\text{l}}) \times {\text{insulin}}\;(\mu {\text{U/ml}})/22.5$$

(Matthews et al. 1985) and

4 $$1/(\log ({\text{insulin}}\,[{\text{mU/l}}] ) + \log ({\text{glucose}}\,[{\text{mg/dl}}] ))$$

(Katz et al. 2000).

### Correlation analyses

Data were visualized in a correlation matrix to facilitate variable selection using imDEV (Grapov and Newman 2012). Variables that are included in this manuscript were those that were statistically significant (*P* < 0.05) and biologically relevant based on available literature. While there were many significant correlations observed within the data set between amino acids and other measured variables, for simplicity we only present here those that helped to guide an explanation of the observed biological phenotype in our study (Table 3). Correlations were calculated by linear regression. Amino acid and fatty acid intake was calculated by multiplying the feed intake of each animal by the quantity of amino acid and fatty acid, respectively, in the diet. Because of differences in feed intake, data that were regressed against amino acid intake (i.e., body fat mass and plasma lipids) were normalized to energy intake prior to regression analysis. Significance was noted at *P* < 0.05 using critical values for Pearson’s correlation coefficient (*df* = 28).

**Table 3:** The strongest and most biologically relevant correlations found by linear regression

	Equation	*R* ^2^	*P* value
Correlations between AA intake and total fat mass (normalized to energy intake)			
Taurine	*y* = −1.100*x* + 1.816	0.4296	<0.0001
Gly	*y* = −1.256*x* + 2.587	0.4113	<0.0001
Met	*y* = 5.952*x* − 1.196	0.4864	<0.0001
Trp	*y* = 12.07*x* − 0.399	0.4048	<0.0001
EAA	*y* = 0.3505*x* − 0.749	0.4157	<0.0001
BCAA	*y* = 0.8298*x* − 0.791	0.4141	<0.0001
Total AA	*y* = 0.1905*x* − 1.603	0.3903	=0.0001
Correlations between AA intake and HDL/total cholesterol (norm. to energy intake)			
Taurine	*y* = 0.1044*x* + 0.2535	0.4694	<0.0001
Gly	*y* = 0.1057*x* + 0.1925	0.3564	<0.0003
Total AA	*y* = −0.0242*x* + 0.6754	0.7676	<0.0001
BCAA	*y* = −0.1001*x* + 0.5585	0.7364	<0.0001
EAA	*y* = −0.0422*x* + 0.5528	0.7358	<0.0001
Trp	*y* = −1.439*x* + 0.5085	0.7027	<0.0001
Met	*y* = −0.6357*x* + 0.5707	0.6781	<0.0001

Total fat mass and HDL-to-total-cholesterol ratio (HDL/total cholesterol) were normalized to energy intake

*EAA* sum of essential amino acids, *BCAA* sum of branched-chain amino acids (Leu, Ile and Val), *total AA* total sum of amino acids

### Statistical analysis

All data are presented as group mean and standard error of the mean. The data were subjected to Analysis of Variance after homogeneity of the variances was confirmed by Levene’s test. Differences between group means were considered statistically significant at *P* < 0.05. Statistical analyses were performed using MiniTab version 16.1 (Minitab Ltd., Coventry, UK) and GraphPad Prism 6 (GraphPad Software Inc., La Jolla, CA, USA). Data from mice fed the low-fat reference diet were not included in the statistical analyses.

## Results

### Reduced body mass gain and energy intake in crab and scallop-fed mice

**Fig. 1:**
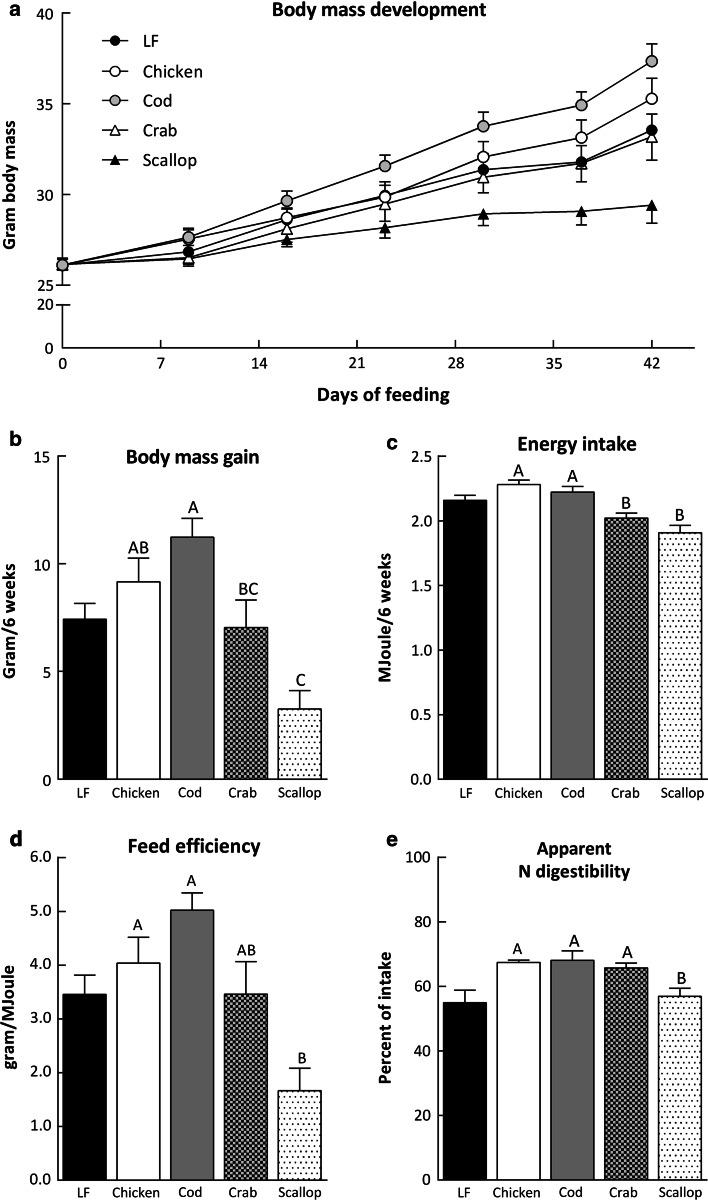
Effect of different protein sources on body mass and feed utilization **a** body mass development, **b** body mass gain, **c** energy intake and **d** feed efficiency in male C57BL/6J mice fed the experimental diets for 6 weeks. **e** Apparent nitrogen digestibility evaluated over 48 h after 4 weeks of feeding (Expt. 1). The data represent group mean values (*n* = 8) ± standard error. Data were analyzed by one-way analysis of variance followed by Tukey’s pair-wise comparisons. Mean values that do not share a letter are significantly different (*P* < 0.05)

During 6 weeks on the experimental diets scallop-fed mice gained significantly less body mass than chicken and cod-fed mice, whereas cod-fed mice gained significantly more body mass than crab and scallop-fed mice (Fig. 1a, b, *P* < 0.001). Chicken and cod-fed mice ingested significantly more energy than crab and scallop-fed mice (Fig. 1c, *P* < 0.001). The feed efficiency, as defined in Eq. 1, was significantly higher in chicken and cod-fed mice than in scallop-fed mice (Fig. 1d, *P* < 0.001). Similar results with regards to body mass gain, energy intake and feed efficiency were obtained during the parallel study (Expt. 1, data not shown) indicating that the results are reproducible.

### Lower apparent nitrogen digestibility in scallop-fed mice

The dietary intake of fat and nitrogen reflected the differences in feed intake and was thus lower in scallop-fed compared to chicken and cod-fed mice (*P* = 0.001 and *P* = 0.004, respectively). No differences were detected in the excretion of nitrogen between groups, while the excretion of fat was significantly lower in scallop-fed mice (*P* = 0.038) compared to chicken-fed mice. Based on intake and excretion, as defined in Eq. 2, no differences in apparent fat digestibility were observed between groups, whereas the apparent nitrogen digestibility was significantly lower in scallop-fed mice than in chicken, cod and crab-fed mice (Fig. 1e, *P* = 0.003, Expt. 1).

### Reduced body mass gain in scallop-fed mice caused by reduced fat accretion

**Fig. 2:**
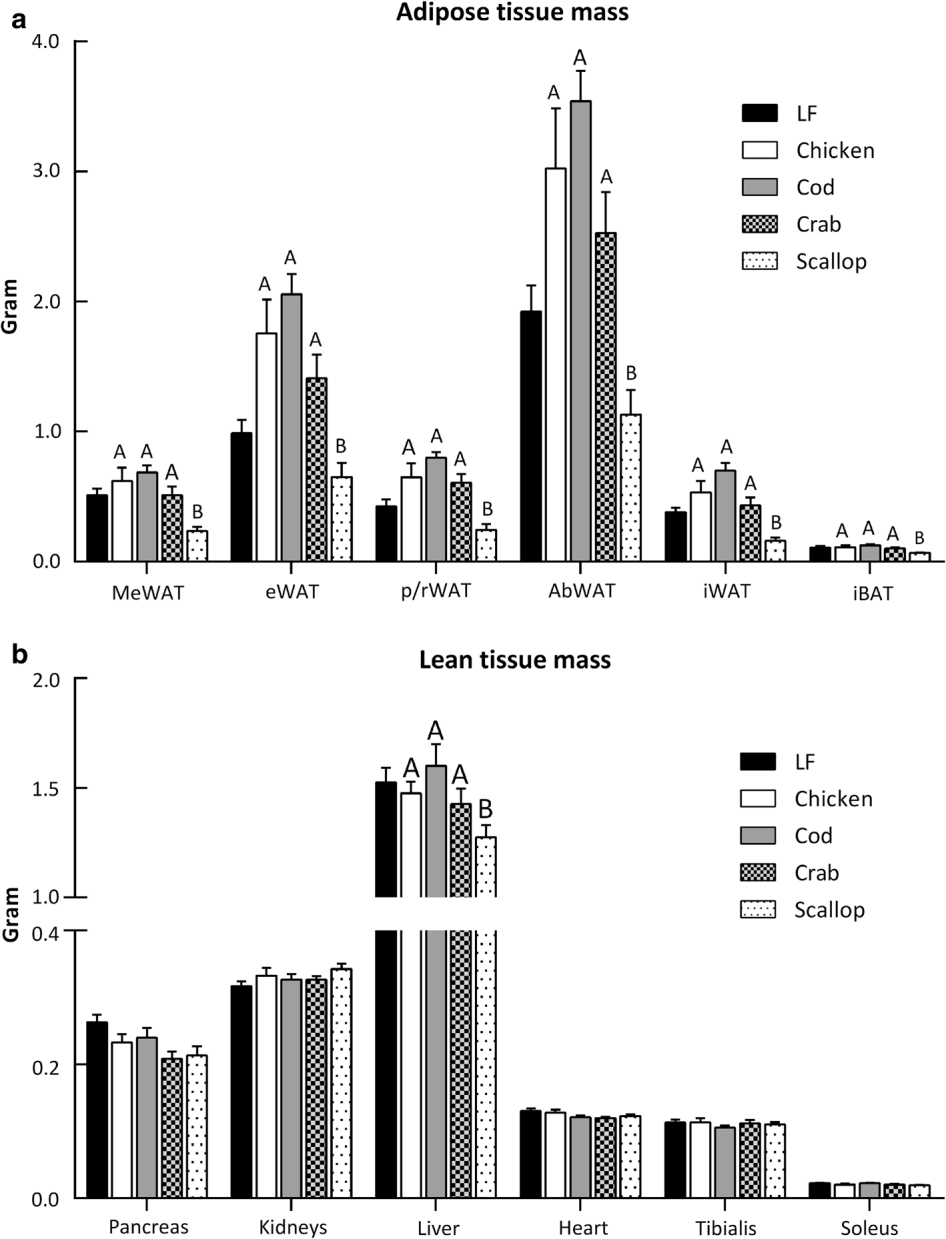
Mass of tissues dissected from male C57BL/6J mice fed the experimental diets for 6 weeks **a** mesenteric white adipose tissue (MeWAT), epididymal white adipose tissue (eWAT), perirenal and retroperitoneal white adipose tissue (p/rWAT), sum of the abdominal fat depots MeWAT, eWAT and p/rWAT (AbWAT), inguinal white adipose tissue (iWAT), interscapular brown adipose tissue (iBAT). **b** Heart, tibialis, soleus, liver, pancreas and kidneys. The data represent group mean values (*n* = 8) ± standard error. Data were analyzed by one-way analysis of variance followed by Tukey’s pair-wise comparisons. Mean values that do not share a letter are significantly different (*P* < 0.05)

Consistent with the reduced body mass gain, scallop-fed mice had reduced visceral and subcutaneous fat depots; MeWAT (*P* < 0.001), eWAT (*P* < 0.001), p/rWAT (*P* < 0.001), iWAT (*P* < 0.001) and iBAT (*P* = 0.001) compared to chicken, cod and crab-fed mice (Fig. 2a). No significant differences were observed in lean tissue mass, i.e., tibialis, soleus and heart or in the mass of pancreas and kidneys between the groups, but the liver weighed less in scallop-fed mice (Fig. 2b, *P* = 0.028) than in chicken, cod and crab-fed mice.

### Improved plasma metabolite profiles in scallop-fed mice

**Fig. 3:**
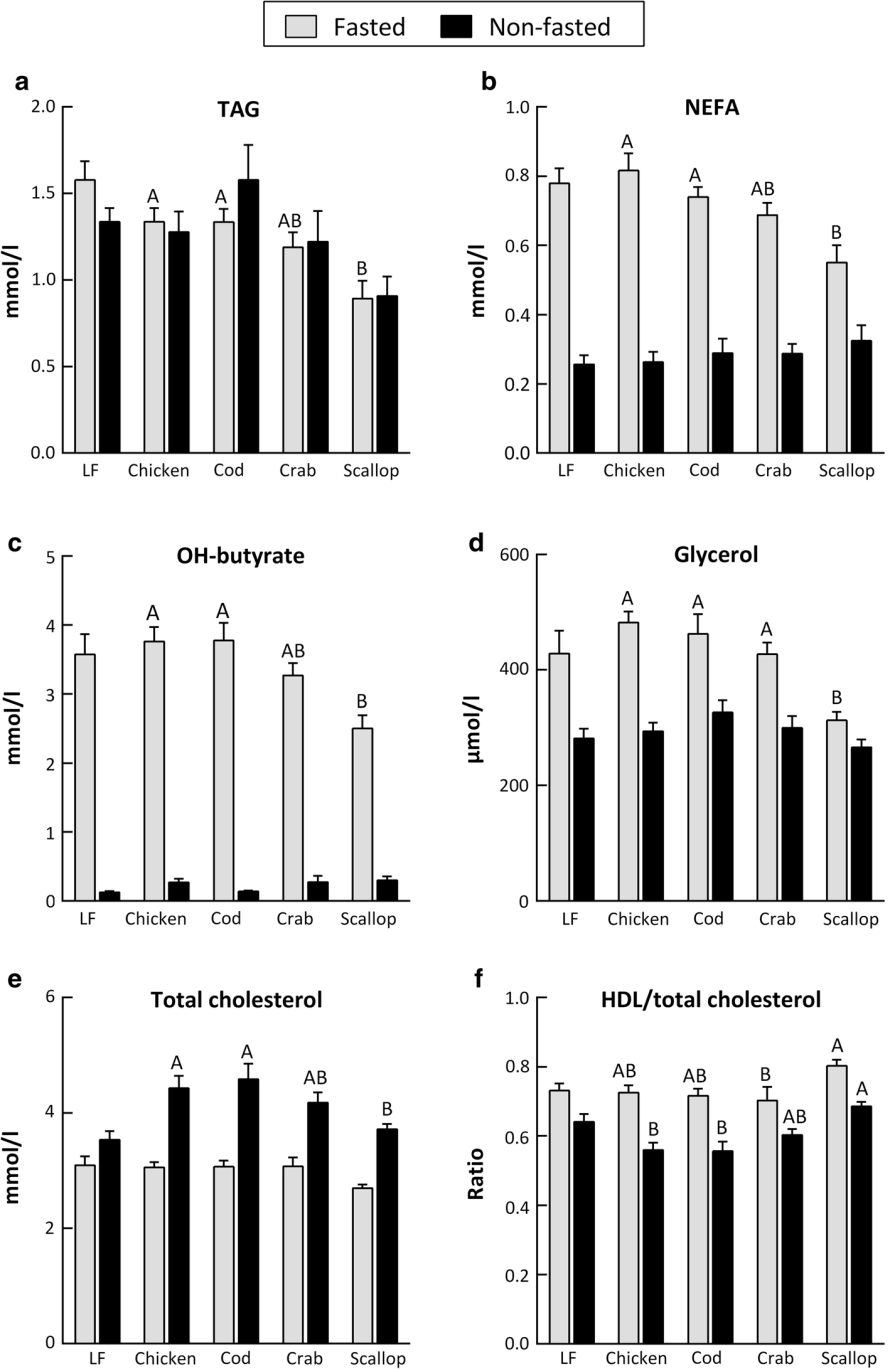
Metabolites in plasma collected in fasted and non-fasted state (Expt. 1 and 2, respectively) at the termination of male C57BL/6J mice following 6 weeks feeding **a** triacylglycerides (TAG), **b** non-esterified fatty acids (NEFA), **c** hydroxy-butyrate (OH-butyrate), **d** glycerol, **e** total cholesterol, **f** HDL-to-total-cholesterol ratio. Data represents group mean values (*n* = 7–8) ± standard error. Data for metabolites in fasted and non-fasted state was analyzed separately by one-way analysis of variance followed by Tukey’s pair-wise comparisons. Mean values that do not share a letter are significantly different (*P* < 0.05)

Fasted (Expt. 1) and non-fasted (Expt. 2) levels of plasma metabolites are shown in Fig. 3 and Supplemental Table 2 (Online Resource 2). Plasma TAG (Fig. 3a, *P* = 0.004), NEFA (Fig. 3b, *P* = 0.001) and OH-butyrate (Fig. 3c, *P* = 0.001) were significantly lower in the fasted state in scallop-fed mice than in chicken and cod-fed mice. Furthermore, in the fasted state plasma glycerol was lower in scallop-fed mice than in chicken, cod and crab-fed mice (Fig. 3d, *P* < 0.001). In the non-fasted state, no differences in NEFA, glycerol and OH-butyrate levels were seen between the groups, but TAG tended to be lower in scallop-fed mice (*P* = 0.057). Plasma total cholesterol (Fig. 3e) was significantly lower in scallop-fed mice than in chicken and cod-fed mice in the non-fasted state (*P* = 0.015) and tended to be lower in scallop-fed mice in the fasted state (*P* = 0.066). The ratio of HDL-to-total-cholesterol (Fig. 3f) was higher in scallop-fed mice than in crab-fed mice in the fasted state (*P* = 0.044) and higher in scallop-fed mice than in chicken and cod-fed mice in the non-fasted state (*P* = 0.001). No significant differences between groups were observed for plasma levels of HDL-cholesterol, LDL-cholesterol, glucose, lactate, total bile acids and alanine aminotransferase, neither in the fasted nor in the non-fasted state, but lactate tended to be lower in scallop-fed mice in the fasted state (*P* = 0.062) and LDL-cholesterol tended to be lower in scallop-fed mice in the non-fasted state (*P* = 0.086) (Supplemental Table 2). No differences were seen in fasted plasma insulin levels between groups. Based on overnight fasted glucose and insulin HOMA-IR and QUICKI scores were calculated, as defined in Eqs. 3 and 4, respectively, but no significant differences were found between the groups (Supplemental Table 2).

### Negative correlations between dietary taurine and glycine intake and total fat mass

To identify factors that might contribute to the observed diet-induced differences in body mass, fat accretion, feed efficiency, apparent nitrogen digestibility and plasma lipid profiles data were visualized in a correlation matrix. The strongest and most biologically relevant correlations are shown in Table 3. The analyses showed highly significant, strong positive correlations between total AA intake and total fat mass normalized to energy intake (*R* ^2^ = 0.39, *P* < 0.0001). Positive correlations of comparable magnitudes were found between fat mass and branched-chain amino acids (BCAA, *R* ^2^ = 0.41, *P* < 0.0001) and essential amino acids (EAA, *R* ^2^ = 0.41, *P* < 0.0001). These observations were expected as AA ingestion reflects feed intake and thereby energy intake; however, after normalizing for energy intake, these correlations were still significant. Of all the AAs intake of methionine was found to positively correlate the strongest with total fat mass (*R* ^2^ = 0.48, *P* < 0.0001), whereas the correlation with tryptophan stands out by having a remarkably high regression coefficient (12.07*x*, *R* ^2^ = 0.40, *P* < 0.0001). While intake of all other AAs correlated positively to total fat mass, strong negative correlations between the consumption of taurine (*R* ^2^ = 0.42, *P* < 0.0001) and glycine (*R* ^2^ = 0.41, *P* < 0.0001) and total fat mass were observed.

### Positive correlations between taurine and glycine intake and improved plasma lipid profile

While dietary intake of most AAs did not correlate significantly with plasma TAG and NEFA, dietary taurine and glycine intake correlated negatively with plasma TAG (*R* ^2^ = 0.16, *P* = 0.032 and *R* ^2^ = 0.21, *P* = 0.011, respectively) and NEFA (*R* ^2^ = 0.18, *P* = 0.019 and *R* ^2^ = 0.17, *P* = 0.019, respectively) in the fasted state. No significant correlations were found between intake of any AAs and total plasma cholesterol. However, we observed negative correlations between intake of all AAs and the ratio of HDL-to-total-cholesterol, except taurine (*R* ^2^ = 0.46, *P* < 0.0001) and glycine (*R* ^2^ = 0.35, *P* < 0.0003) which were positively correlated with HDL-to-total-cholesterol ratios (Table 3).

## Discussion

### Reduced energy intake in crab and scallop-fed mice

It is likely that the reduced energy intake seen in crab and scallop-fed mice, compared to chicken and cod-fed mice, contributed to the altered phenotypes. The underlying mechanism for the reduced energy intake is not established, but the taurine content was high in these diets, particularly in the scallop diet. Interestingly, a single intracerebroventricular injection of taurine was shown to enhance the anorexigenic effect of insulin in hypothalamus and to reduce energy intake in rats (Solon et al. 2012). It has been reported that taurine enters the brain after oral administration (Urquhart et al. 1974) and intraperitoneal injection (Peck Jr and Awapara 1967) in the rat, but it is still debated whether and to what extent orally and peripherally administered taurine is able to traverse the blood brain barrier (Ripps and Shen 2012). Therefore, it is also debatable whether the effects of taurine seen after an intracerebroventricular injection are relevant in the physiological setting. However, it was recently reported that in male C57BL/6J mice taurine supplementation in the drinking water enhanced hypothalamic insulin action which in turn decreased energy intake and prevented high-fat diet-induced obesity (Camargo et al. 2013). Furthermore, in humans a positive correlation between satiety and elevated postprandial blood taurine concentrations after consumption of test meals was reported (Veldhorst et al. 2009). Thus, the high taurine content in the crab diet and especially in the scallop diet might have contributed to the reduced energy intake but further analyses are needed to establish whether taurine affected satiety and thereby decreased energy intake in crab and scallop-fed mice in this experiment. Even though the energy intake was lower in both crab and scallop-fed mice, the body and fat mass gain was significantly reduced in scallop-fed mice only. Together with the reduced feed efficiency and apparent nitrogen digestibility this suggests that metabolism was affected in scallop-fed mice.

### Unlike all other amino acids, the intake of taurine and glycine correlated negatively with fat mass

Although the experimental diets were isonitrogenous, the scallop diet had slightly lower total AA content, including EAA and BCAA, than the chicken, cod and crab diets. Importantly, except slightly decreased liver mass in scallop-fed mice no differences were seen in lean body mass between groups, effectively ruling out impaired protein synthesis as the explanation of the reduced body mass gain in scallop-fed mice. We hypothesize that the reduced liver mass in scallop-fed mice may be associated with the observed reduced level of circulating lipids, i.e., fasted plasma TAG and NEFA and non-fasted plasma total cholesterol. Decreased storage of hepatic lipids may have contributed to the reduced liver mass in scallop-fed mice, but further studies are needed to confirm this. Our correlation analyses showed highly significant, strong positive correlations between dietary intake of all AAs, except taurine and glycine, and total fat mass, even after correcting for differences in energy intake. Dietary methionine intake was found to positively correlate the strongest with fat mass. Interestingly, methionine restriction has been shown to prevent diet-induced obesity in rodents (Malloy et al. 2006; Hasek et al. 2010; Perrone et al. 2013; Ables et al. 2012). Therefore, even though the mice’s dietary sulfur-amino acid requirements (Nutrient Requirements of Laboratory Animals, Fourth Revised Edition, 1995) were fulfilled by the experimental diets, the dietary methionine load might have influenced the growth and adiposity in the present study. Highly significant negative correlations were found between the consumption of taurine as well as glycine and fat mass in male C57BL/6J mice. This is consistent with other studies showing that taurine supplementation reduced body weight in human subjects (Zhang et al. 2004) and fat mass in rodents (Nardelli et al. 2011; Tsuboyama-Kasaoka et al. 2006), and that glycine supplementation reduced adipocyte size and visceral fat mass in rats (El Hafidi et al. 2004). Scallop-fed mice were found to have decreased fasting plasma TAG, NEFA, glycerol and OH-butyrate and a tendency (*P* = 0.062) towards increased fasting plasma lactate suggesting a shift towards decreased lipid metabolism and increased glucose metabolism. Taurine supplementation has previously been associated with changes in fat metabolism in rodents (Murakami et al. 1998; Tsuboyama-Kasaoka et al. 2006) and with upregulating signaling cascades that increase nutrient utilization and energy expenditure in humans (Yeh et al. 2011) and mice (Tsuboyama-Kasaoka et al. 2006) while decreasing fed state energy expenditure and glucose oxidation rate in diabetic rats (Harada et al. 2004). Similarly, glycine supplementation has been shown to increase energy expenditure in mice (Alarcon-Aguilar et al. 2008; Almanza-Perez et al. 2010). We have previously shown that a diet with fish protein hydrolysate, rich in taurine and glycine, elevated plasma bile acid concentration and reduced adiposity in rats (Liaset et al. 2009, 2011). It is suggested that bile acids increase energy expenditure, possibly through activation of farnesoid-X-receptor and TGR5, which both affect metabolism and energy expenditure (Prawitt et al. 2011). However, despite the large differences in taurine and glycine intake, no differences in fasted or non-fasted plasma total bile acid concentration were observed in mice from the present study. Thus, it is unlikely that the alteration of body and fat mass gain was affected by bile acids in the present study. Taken together, intake of taurine and glycine correlated negatively with body and fat mass gain possibly due to changes in metabolism and energy substrate utilization, but further experiments are needed to identify the mechanisms behind the observed differences.

### Improved plasma lipid profile correlate with taurine and glycine intake

The scallop-fed mice had improved plasma lipid profiles with lower plasma TAG, NEFA and total cholesterol and increased HDL-to-total-cholesterol ratio, compared to mice fed the other diets. Due to a small contribution from the protein sources the seafood diets contained low levels of the marine n-3 long-chain PUFAs eicosapentaenoic acid (EPA) and docosahexaenoic acid (DHA), Supplemental Table 1 (Online Resource 1), that hypothetically could contribute to the reduced body mass gain and improved plasma lipid profiles (Alvheim et al. 2012; Frøyland et al. 1997; Janovska et al. 2013; Madsen et al. 1998) seen in scallop-fed mice. However, our analyses revealed no significant correlation between EPA or DHA intake and body mass gain or plasma lipid profile and consequently, the low doses of n-3 PUFAs are likely to be negligible in the present study. Our correlation analyses not only showed highly significant, strong correlations between especially taurine but also glycine intake and plasma HDL-to-total-cholesterol ratio. These findings are in line with previous studies showing that taurine supplementation improved serum lipid profile in overweight subjects (Zhang et al. 2004) and improved cholesterol profile in rodents (Murakami et al. 1998; Nardelli et al. 2011; Yokogoshi et al. 1999), specifically by decreasing non-HDL cholesterols (Chen et al. 2012; Murakami et al. 2002; Sugiyama et al. 1989) and that glycine supplementation reduced plasma lipids in sucrose-fed rats (El Hafidi et al. 2004). The increased HDL-to-total-cholesterol ratio suggests increased reverse cholesterol transport or clearance, and taurine has in fact been shown to upregulate key genes involved in reverse cholesterol transport in cultured cells in vitro (Hoang et al. 2012), and to upregulate LDL receptor binding and activity in vitro (Stephan et al. 1987) and in the liver of hamsters (Murakami et al. 2002). The reduced plasma total cholesterol level in scallop-fed mice may be associated with the lower cholesterol content in the scallop diet compared to the chicken, cod and crab diets (Table 1). However, this does not explain the increased plasma HDL-to-total-cholesterol ratio in the scallop-fed mice. Moreover, the LF diet had very low cholesterol content (0.06 g/kg). If LF-fed mice are included in the statistical analyses no statistically significant differences were found in plasma total cholesterol between any of the groups in the fasted state. In the non-fasted state LF-fed mice had decreased plasma total cholesterol compared to cod and chicken-fed mice (*P* = 0.001), while cod-fed mice had higher plasma total cholesterol levels than LF and scallop-fed mice (*P* = 0.001). No differences were found in HDL-to-total-cholesterol ratio between LF-fed mice and chicken, cod or crab-fed mice neither in the fasted nor the non-fasted state, while scallop-fed mice maintained an increased HDL-to-total-cholesterol ratio compared to chicken and cod-fed mice in the non-fasted state (*P* = 0.001) and tended to have increased HDL-to-total-cholesterol ratio compared to crab-fed mice in the fasted state (*P* = 0.059). Therefore, our data indicated that the lower dietary cholesterol concentration did not by itself explain the higher HDL-to-total-cholesterol ratio observed in scallop-fed mice. Taken together, the improved HDL-to-total-cholesterol ratio in scallop-fed mice did not correlate with the differences in lipid composition between the diets but did correlate significantly with dietary taurine and glycine intake.

Apart from the described highly significant linear correlations with intake of taurine and glycine, we cannot exclude that other nutritional factors in the scallop protein might also have contributed to the reduced body mass gain and improved plasma lipid profile, but further analyses are needed to identify other contributing nutrients. Furthermore, non-linear correlations that we are unable to identify in the present study may exist between consumed nutrients and body mass gain and plasma lipid profile.

In conclusion, intake of scallop muscle as the sole dietary protein source completely prevented high-fat, high-sucrose-induced body mass gain and fat accretion without affecting lean body mass. Furthermore, scallop feeding improved plasma lipid profile in C57BL/6J mice compared to mice fed diets with protein from chicken, cod or crab. Correlation analyses revealed strong, highly significant inverse correlations between intake of taurine and glycine and body fat mass, as well as strong, highly significant correlations between glycine and especially taurine intake and improved plasma lipid profiles. Changes in satiety, energy expenditure, energy substrate utilization and cholesterol metabolism cannot be ruled out as significant contributing factors in the present study, but further experiments are needed to explore these variables fully.

## Electronic supplementary material

Below is the link to the electronic supplementary material.

Supplementary material 1 (PDF 65 kb)

Supplementary material 2 (PDF 75 kb)
