# Supplementary material for: Scallop protein with endogenous high taurine and glycine content prevents high-fat, high-sucrose-induced obesity and improves plasma lipid profile in male C57BL/6J mice
Source: Amino Acids. 2014 Mar 23;46(7):1659–71. doi: 10.1007/s00726-014-1715-1 (PMC4055845; doi:10.1007/s00726-014-1715-1)
Supplement: Supplementary file 1 — Supplementary material 1 (PDF 65 kb) [file 726_2014_1715_MOESM1_ESM.pdf]

Article title

Scallop protein with endogenous high taurine and glycine content prevents high fat, high sucrose-induced obesity and improves plasma lipid profile in male C57BL/6J mice

Journal name

Amino Acids

Author names

Hanne Sørup Tastesen[1,2], Alison H. Keenan[3], Lise Madsen[1,2], Karsten Kristiansen[1] and Bjørn Liaset[2]

Author affiliations

[1] University of Copenhagen, Department of Biology, Ole Maaløes Vej 5, 2200 Copenhagen, Denmark

[2] National Institute of Nutrition and Seafood Research, Postbox 2029 Nordnes, 5817 Bergen, Norway

[3] Massachusetts General Hospital, Center for Computational and Integrative Biology, 185 Cambridge Street, Boston, MA 02114, USA

Corresponding authors

Bjørn Liaset; Bjorn.Liaset@nifes.no

Karsten Kristiansen; Karsten.Kristiansen@bio.ku.dk

Supplemental Table 1 Composition of fatty acids in the diets

(g/kg)	LF	Chicken	Cod	Crab	Scallop
10:0	< 0.01	0.20	0.10	0.10	0.10
12:0	< 0.01	0.20	0.10	0.10	0.10
14:0	0.20	2.85	2.75	2.75	2.70
15:0	< 0.01	0.20	0.20	0.20	0.20
16:0	6.00	68.05	66.10	65.40	64.65
17:0	0.10	0.95	0.90	0.90	0.90
18:0	2.85	29.90	28.75	28.40	28.20
20:0	0.10	0.70	0.80	0.75	0.85
22:0	0.10	0.15	0.30	0.15	0.30
14:1n-9	< 0.01	0.15	0.10	0.10	0.10
16:1n-7	0.32	4.23	4.10	4.25	4.23
16:1n-9	0.10	0.70	0.60	0.70	0.70
18:1n-7	0.70	6.20	5.85	6.05	5.95
18:1n-9	10.70	132.70	127.25	126.75	125.15
20:1n-9	0.10	1.60	1.45	1.50	1.65
18:2n-6	16.10	125.05	121.40	120.25	119.00
20:2n-6	0.10	0.75	0.85	0.90	1.00
18:3n-3	1.90	2.95	2.75	2.70	2.60
20:3n-3	< 0.01	0.20	0.15	0.15	0.15
20:3n-6	< 0.01	< 0.01	< 0.01	0.10	< 0.01
16:4n-3	< 0.01	< 0.01	0.45	0.20	< 0.01
20:4n-6	0.10	0.65	0.55	0.65	0.60
22:4n-6	< 0.01	0.15	0.10	< 0.01	0.10
20:5n-3[a]	< 0.01	< 0.01	0.85	1.40	1.35
22:5n-3	< 0.01	< 0.01	< 0.01	< 0.01	0.10
22:6n-3[b]	< 0.01	< 0.01	1.85	0.95	0.90

[a] eicosapentaenoic acid

[b] docosahexaenoic acid
