# Supplementary material for: Scallop protein with endogenous high taurine and glycine content prevents high-fat, high-sucrose-induced obesity and improves plasma lipid profile in male C57BL/6J mice
Source: Amino Acids. 2014 Mar 23;46(7):1659–71. doi: 10.1007/s00726-014-1715-1 (PMC4055845; doi:10.1007/s00726-014-1715-1)
Supplement: Supplementary file 2 — Supplementary material 2 (PDF 75 kb) [file 726_2014_1715_MOESM2_ESM.pdf]

Article title

Scallop protein with endogenous high taurine and glycine content prevents high fat, high sucrose-induced obesity and improves plasma lipid profile in male C57BL/6J mice

Journal name

Amino Acids

Author names

Hanne Sørup Tastesen[1,2], Alison H. Keenan[3], Lise Madsen[1,2], Karsten Kristiansen[1] and Bjørn Liaset[2]

Author affiliations

[1] University of Copenhagen, Department of Biology, Ole Maaløes Vej 5, 2200 Copenhagen, Denmark

[2] National Institute of Nutrition and Seafood Research, Postbox 2029 Nordnes, 5817 Bergen, Norway

[3] Massachusetts General Hospital, Center for Computational and Integrative Biology, 185 Cambridge Street, Boston, MA 02114, USA

Corresponding authors

Bjørn Liaset; Bjorn.Liaset@nifes.no

Karsten Kristiansen; Karsten.Kristiansen@bio.ku.dk

Supplemental Table 2 Measured plasma metabolites and calculated HOMA-IR and QUICKI scores

	State	LF	Chicken	Cod	Crab	Scallop	P-value
TAG (mmol/l)	*Fasted*	1.58 ± 0.11	^{A}1.34 ± 0.08	^{A}1.33 ± 0.08	^{AB}1.19 ± 0.09	^{B}0.89 ± 0.10	0.004
	Non-fasted	0.91 ± 0.13	1.27 ± 0.13	1.58 ± 0.20	1.22 ± 0.19	0.91 ± 0.11	0.057
NEFA (mmol/l)	*Fasted*	0.78 ± 0.04	^{A}0.82 ± 0.05	^{A}0.74 ± 0.03	^{AB}0.69 ± 0.04	^{B}0.55 ± 0.05	0.001
	Non-fasted	0.26 ± 0.03	0.26 ± 0.03	0.29 ± 0.04	0.29 ± 0.03	0.32 ± 0.04	0.335
Glycerol (mmol/l)	*Fasted*	0.43 ± 0.04	^{A}0.48 ± 0.02	^{A}0.46 ± 0.03	^{A}0.43 ± 0.02	^{B}0.31 ± 0.01	0.001
	Non-fasted	0.28 ± 0.02	0.29 ± 0.02	0.33 ± 0.02	0.30 ± 0.02	0.27 ± 0.01	0.323
OH-butyrate (mmol/l)	*Fasted*	3.57 ± 0.29	^{A}3.76 ± 0.21	^{A}3.78 ± 0.26	^{AB}3.27 ± 0.18	^{B}2.50 ± 0.19	0.001
	Non-fasted	0.13 ± 0.02	0.27 ± 0.05	0.29 ± 0.16	0.28 ± 0.09	0.30 ± 0.05	0.260
Total cholesterol (mmol/l)	*Fasted*	3.09 ± 0.16	3.06 ± 0.09	3.07 ± 0.10	3.07 ± 0.16	2.70 ± 0.06	0.066
	Non-fasted	3.54 ± 0.16	^{A}4.43 ± 0.23	^{A}4.59 ± 0.27	^{AB}4.18 ± 0.19	^{B}3.72 ± 0.09	0.015
LDL-cholesterol (mmol/l)	*Fasted*	0.57 ± 0.04	0.56 ± 0.04	0.53 ± 0.02	0.60 ± 0.07	0.45 ± 0.02	0.119
	Non-fasted	0.63 ± 0.04	0.81 ± 0.06	0.78 ± 0.06	0.72 ± 0.05	0.66 ± 0.03	0.086
HDL-cholesterol (mmol/l)	*Fasted*	2.25 ± 0.10	2.22 ± 0.06	2.20 ± 0.09	2.13 ± 0.11	2.16 ± 0.04	0.880
	Non-fasted	2.25 ± 0.07	2.45 ± 0.08	2.50 ± 0.07	2.50 ± 0.06	2.55 ± 0.06	0.944
HDL/total cholesterol	*Fasted*	0.73 ± 0.02	^{AB}0.73 ± 0.02	^{AB}0.72 ± 0.02	^{B}0.70 ± 0.04	^{A}0.80 ± 0.02	0.044
	Non-fasted	0.64 ± 0.02	^{B}0.56 ± 0.02	^{B}0.56 ± 0.03	^{AB}0.60 ± 0.02	^{A}0.69 ± 0.01	0.001
ALAT (U/l)	*Fasted*	23.33 ± 2.87	31.86 ± 4.86	29.00 ± 3.25	28.00 ± 3.64	20.71 ± 1.11	0.175
	Non-fasted	27.75 ± 3.60	21.50 ± 1.05	25.00 ± 2.67	18.75 ± 1.26	19.50 ± 2.13	0.064
Lactate (mmol/l)	*Fasted*	1.53 ± 0.03	1.94 ± 0.11	1.70 ± 0.10	2.25 ± 0.23	2.13 ± 0.11	0.062
	Non-fasted	4.27 ± 0.42	3.80 ± 0.44	4.01 ± 0.40	3.95 ± 0.39	3.47 ± 0.29	0.866
Total bile acids (µmol/l)	*Fasted*	2.20 ± 0.52	1.77 ± 0.42	1.45 ± 0.22	1.60 ± 0.39	2.73 ± 0.68	0.202
	Non-fasted	3.86 ± 0.48	1.30 ± 0.44	1.71 ± 0.35	1.49 ± 0.40	2.16 ± 0.33	0.381
Glucose (mmol/l)	*Fasted*	6.52 ± 0.51	9.63 ± 0.52	8.96 ± 0.53	9.07 ± 0.72	10.18 ± 0.91	0.566
	Non-fasted	16.32 ± 0.78	16.17 ± 0.44	16.46 ± 0.90	14.80 ± 0.61	16.55 ± 0.40	0.214
Insulin (pmol/l)	*Fasted*	15.12 ± 6.54	19.95 ± 4.80	23.75 ± 4.34	37.47 ± 6.60	20.78 ± 5.35	0.101
HOMA-IR	*Fasted*	0.91 ± 0.23	1.46 ± 0.29	1.61 ± 0.26	2.19 ± 0.40	1.41 ± 0.42	0.459
QUICKI	*Fasted*	0.43 ± 0.02	0.37 ± 0.01	0.37 ± 0.01	0.36 ± 0.02	0.39 ± 0.02	0.658

Data represent group means (n=6-8) ± standard error. Data was analysed by one-way analysis of variance followed by Tukey's pairwise comparisons. Means that do not share a letter are significantly different (P<0.05). HOMA-IR: Homeostasis Model of Assessment-Insulin Resistance, QUICKI: Quantitative Insulin Sensitivity Check Index.
